# National Convention of Physicians

**Published:** 1845-07

**Authors:** 


### NATIONAL CONVENTION OF PHYSICIANS.

In the transactions of the New York State Medical Society, we find the following in reference to a National Convention.

The following preamble and resolution were presented by Dr. Davis and adopted, and Drs. Davis, McNaughton, and the Secretary, Dr. Van Buren, were appointed a committee to carry out the proposed measure.

*Whereas*, It is believed that a National Convention would be conducive to the elevation of the standard of medical education in the United States, and

*Whereas*, There is no mode of accomplishing so desirable an object, without concert of action on the part of the medical societies, colleges, and institutions of all the states. Therefore,

***Resolved***, That the New York State Medical Society earnestly recommend a national convention of delegates from medical societies and colleges in the whole Union—to convene in the city of New York, on the first Tuesday in May, in the year 1846, for the purpose of adopting some concerted action on the subject set forth in the foregoing preamble.

This is an important movement, and one which we have long desired to see. We call to it the early attention of those who honor us by reading the pages of the Examiner, that they may reflect and be prepared to act upon the subject when the proper time arrives. On another page we have hinted at some of the benefits that accrue from such association, and we will now only add the expression of our conviction, that whatever it is desirable to accomplish by way of reform in the practice of medicine, must be achieved by the moral influence of the members of the profession, and not by legislative enactments; and this moral influence can be rendered vastly more effective by comparison of sentiment and unity of action among those who feel an interest in the subject.

